# Tumour dose response to the antivascular agent ZD6126 assessed by magnetic resonance imaging

**DOI:** 10.1038/sj.bjc.6600926

**Published:** 2003-05-13

**Authors:** S P Robinson, D J O McIntyre, D Checkley, J J Tessier, F A Howe, J R Griffiths, S E Ashton, A J Ryan, D C Blakey, J C Waterton

**Affiliations:** 1 Cancer Research UK Biomedical Magnetic Resonance Research Group, St George's Hospital Medical School, London SW17 ORE, UK; 2 Department of Enabling Science and Technology, AstraZeneca, Alderley Park, Macclesfield, Cheshire SK10 4TG, UK; 3 Department of Cancer and Infection Research, AstraZeneca, Alderley Park, Macclesfield, Cheshire SK10 4TG, UK

**Keywords:** ZD6126, vascular targeting agents, MRI, tumour perfusion, response biomarker

## Abstract

ZD6126 is a vascular targeting agent that disrupts the tubulin cytoskeleton of proliferating neo-endothelial cells. This leads to the selective destruction and congestion of tumour blood vessels in experimental tumours, resulting in extensive haemorrhagic necrosis. In this study, the dose-dependent activity of ZD6126 in rat GH3 prolactinomas and murine RIF-1 fibrosarcomas was assessed using two magnetic resonance imaging (MRI) methods. Dynamic contrast-enhanced (DCE) MRI, quantified by an initial area under the time–concentration product curve (IAUC) method, gives values related to tumour perfusion and vascular permeability. Multigradient recalled echo MRI measures the transverse relaxation rate *T*_2_^*^, which is sensitive to tissue (deoxyhaemoglobin). Tumour IAUC and *R*_2_^*^ (=1/*T*_2_^*^) decreased post-treatment with ZD6126 in a dose-dependent manner. In the rat model, lower doses of ZD6126 reduced the IAUC close to zero within restricted areas of the tumour, typically in the centre, while the highest dose reduced the IAUC to zero over the majority of the tumour. A decrease in both MRI end points was associated with the induction of massive central tumour necrosis measured histologically, which increased in a dose-dependent manner. Magnetic resonance imaging may be of value in evaluation of the acute clinical effects of ZD6126 in solid tumours. In particular, measurement of IAUC by DCE MRI should provide an unambiguous measure of biological activity of antivascular therapies for clinical trial.

A functioning vascular network is a prerequisite for tumour growth, providing tumour cells with a nutritive blood supply and facilitating the removal of toxic waste products of metabolism. It is well known that the architecture of tumour blood vessels differs considerably from that of normal tissues (Denekamp, 1993). For example, tumour blood vessels contain a significant component of proliferating, immature endothelium, in contrast to the quiescent endothelium found in most normal adult tissues. The continued expression of proangiogenic growth factors in tumours can result in an endothelial cell proliferation rate 35-fold higher than that of normal tissues (Denekamp and Hobson, 1982). Novel anticancer therapies are being developed to exploit differences between normal and tumour endothelium, with the aim of selectively targeting the destruction of tumour endothelium while leaving normal blood vessels relatively unaffected (Dark *et al*, 1997). This approach is particularly attractive since the destruction of one tumour blood vessel has the potential to kill many dependent tumour cells (Chaplin and Dougherty, 1999).

ZD6126 is a novel vascular targeting agent that has been shown to have significant antitumour activity against a broad range of human xenografts in rodent model systems, including early-phase lung cancer metastases (Blakey *et al*, 2002; Davis *et al*, 2002; Goto *et al*, 2002). Previous work in mice and rats has shown that ZD6126 is rapidly converted by serum phosphatase activity to *N*-acetylcolchinol (NAC), a tubulin binding agent that inhibits tubulin polymerisation and causes microtubule destabilisation.

Studies of cultured human endothelial cells *in vitro* have demonstrated that NAC disrupts the tubulin cytoskeleton and produces rapid changes in endothelial cell morphology at noncytotoxic concentrations. Moreover, these effects are selective for proliferating compared with confluent endothelial cells. *In vivo*, within an hour of ZD6126 administration, there is evidence of exposure of the basal lamina of tumor blood vessels, with retraction and subsequent loss of endothelial cells (Blakey *et al*, 1999). These early changes are followed by thrombosis and vessel occlusion resulting in extensive central tumour necrosis 24 h after ZD6126 treatment. The vascular targeting activity of ZD6126 was seen at doses 1/8 to 1/16 of the maximum well-tolerated dose, and was selective for tumor blood vessels, with no evidence of similar effects in the vessels of surrounding normal tissues (Blakey *et al*, 2002; Davis *et al*, 2002). A consistent observation from preclinical models is that a thin rim of tumour cells at the edge of the tumour mass survives treatment with ZD6126, presumably because these cells are fed from unaffected vessels in the surrounding normal tissues, although there is no direct evidence for this.

The clinical development of novel antivascular therapies requires the development and validation of quantitative end points that are associated with tumour blood vasculature and its response, and that can be translated to the clinic. Magnetic resonance imaging (MRI) is one approach that affords such biomarkers of response to antivascular therapies, and drug efficacy has been demonstrated in both preclinical (Beauregard *et al*, 2002; Maxwell *et al*, 2002) and clinical studies (Dowlati *et al*, 2002; Galbraith *et al*, 2002). In this study, we describe the use of two MRI techniques for the noninvasive assessment of the *dose-dependent* antivascular activity of ZD6126 on tumour perfusion. In the first study, dose–response to ZD6126 in rat GH3 prolactinomas was determined using dynamic contrast-enhanced (DCE) MRI, in which the tumour uptake of exogenously administered Gd-DTPA-BMA (gadolinium diethylenetriamine penta-acetic acid bismethylamide or gadodiamide) is monitored (Evelhoch, 1999). In a second study, multi-gradient recalled echo (MGRE) MRI was used to assess efficacy of ZD6126 in two rodent tumour models, rat GH3 prolactinomas and murine RIF-1 fibrosarcomas. MGRE MRI allows quantification of the transverse relaxation rate *R*_2_^*^ (=1/*T*_2_^*^), which is dependent on the tissue deoxyhaemoglobin concentration (Abramovitch *et al*, 1999; Kostourou *et al*, 2002).

## METHODS

### Animals and tumours

All experiments were performed in accordance with the local ethical review panel, the UKCCCR guidelines (Workman *et al*, 1998) and the UK Home Office Animals Scientific Procedures Act, 1986. The data were accrued from two rodent tumour models, the rat GH3 prolactinoma grown s.c. in the flanks of female Wistar Furth rats (Prysor-Jones and Jenkins, 1981) and the murine RIF-1 fibrosarcoma grown s.c. in the flanks of female C3 H mice (Twentyman *et al*, 1980). Anaesthesia was induced with a 4 ml kg^−1^(rat) or 10 ml kg^−1^ (mouse) intraperitoneal injection of fentanyl citrate (0.315 mg ml^−1^) plus fluanisone (10 mg ml^−1^) (‘Hypnorm’, Janssen Pharmaceutical Ltd, High Wycombe), midazolam (5 mg ml^−1^) (‘Hypnovel’, Roche, Welwyn Garden City) and water (1 : 1 : 2). This anaesthetic mixture has been shown to have a minimal effect on tumour blood flow (Menke and Vaupel, 1988). Tumour volume was measured using calipers, assuming an ellipsoidal shape.

### Formulation, administration and dosing of ZD6126

ZD6126 (*N*-acetylcolchinol-*O*-phosphate) was formulated in 20% of 5% sodium carbonate and 80% phosphate-buffered saline yielding a clear solution at ∼pH 7, and was rapidly administered intravenously as a bolus injection via a tail vein. Control animals were treated with vehicle alone. Doses of 12.5, 25 or 50 mg kg^−1^ ZD6126 for rats and 100 or 200 mg kg^−1^ ZD6126 for mice were used which, when taking into account the surface law formula, are similar and well-tolerated host dose regimes (Blakey *et al*, 2002).

### Dynamic contrast-enhanced MRI

The dose–response of rat GH3 prolactinomas to ZD6126 was determined using DCE MRI, performed 24 h prior to and 24 h post-treatment with phosphate-buffered saline (*n*=6), 12.5 mg kg^−1^ (*n*=5), 25 mg kg^−1^ (*n*=5) or 50 mg kg^−1^ (*n*=5) ZD6126 administered intravenously. The mean tumour volume for this study was 3.49±0.3 cm^3^ (range 2.54–5.38 cm^3^). Rats were anaesthetised, a butterfly catheter inserted into a tail vein for contrast agent administration, and placed supine into a 63 mm quadrature birdcage coil. Image sets were acquired on a Varian Unity Inova system interfaced to a 4.7 T horizontal magnet from four contiguous 2 mm slices through the tumour and one through the abdomen to provide a muscle tissue reference, using a spin-echo sequence with repetition time TR=120 ms, echo time TE=10 ms and a 128 × 128 matrix, giving an acquisition time of 15.4 s per image set. Prior to DCE MRI, spin-echo images were acquired from the same slices and with the same field of view and spatial resolution as the time course data to provide baseline *T*_1_ data. The images were acquired with TE=120 ms and TR=0.12, 0.5, 2 and 10 s. In some cases, these data were either missing or were too noisy, so mean data from the remainder of the cohort were used. The mean baseline *R*_1_ (=1/*T*_1_) obtained was 0.53±0.07 s^−1^ (mean±s.d.). Since the change in *R*_1_ in well-vascularised pixels was typically three times this value, it is unlikely that serious errors were introduced by the use of this mean value. Five image sets were acquired prior to and 40 sets after bolus injection of 0.1 mmol kg^−1^ gadodiamide (Gd-DTPA-BMA) (Omniscan, Amersham Health, Little Chalfont). The total DCE MR imaging time postinjection was 10 min 15 s.

Data were transferred and processed in software developed by the authors (JJT, DJOM), and running under IDL 5.3/5.4 (Research Systems, Boulder, CO, USA). For each tumour slice, a region-of-interest (ROI) was drawn over the whole tumour, but excluding the surrounding skin/muscle. A similar ROI was drawn over back muscle in the abdominal slice. The five preinjection images were averaged to increase signal-to-noise ratio and subtracted from the postinjection images to give enhancement images. Tumour and muscle *T*_1_ time courses were calculated on a pixel-by-pixel basis from the enhancement and baseline *T*_1_ values and converted to gadodiamide concentrations using the known relaxivity of gadodiamide. The initial area under the time–concentration product curve (IAUC) for the first 150 s after administration of gadodiamide was then integrated, and the tumour data normalised to the median muscle IAUC to account for differences in the arterial input function between animals (Evelhoch, 1999). This methodology has been endorsed for use in studies of antivascular agents (Leach *et al*, 2003). The data were reviewed blind as images and normalised IAUC frequency histograms. Tumour IAUC values greater than the muscle median IAUC were defined as highly enhancing pixels. This threshold was not chosen to distinguish muscle and tumour, but to define regions of tumour that did not enhance significantly. The choice of this cutoff point for the highly enhancing fraction was validated by cumulative histogram analysis of the change in highly enhancing fraction with treatment. Statistical analysis of the changes induced by ZD6126 treatment were based on the change in the proportion of highly enhancing pixels.

After DCE MRI, the tumours were excised, fixed in formal saline, cut in the same plane/orientation as for the MRI, and stained with haematoxylin and eosin. Tissue sections were assessed for necrosis, using a scale from grade 1 (0–10% necrosis) to grade 10 (>90–100% necrosis) (Blakey *et al*, 2002). The necrosis scores represent the median value of five sections for each tumour at each dose level.

### Multi-gradient recalled echo MRI

ZD6126 was administered intravenously to rats bearing GH3 prolactinomas (0, 25 or 50 mg kg^−1^) and mice bearing RIF-1 fibrosarcomas (0, 100 or 200 mg kg^−1^) (*n*=3 per treatment group). The mean tumour volume for the rat GH3 prolactinomas was 3.61±0.41 cm^3^ (range 2.92–4.3 cm^3^), and for the murine RIF-1 fibrosarcomas was 0.7±0.13 cm^3^ (range 0.53–0.91 cm^3^). ^1^H MRI was performed 24 h later at 4.7 T using a 3-turn, 25 mm coil for rats or a 2-turn 12 mm coil for mice. The anaesthetised animals were positioned so that the tumour hung vertically into the radiofrequency coil and covered with a warm water blanket to maintain the core temperature at 37°C. Field homogeneity was optimised by shimming on the water signal for each tumour to a linewidth of 30–50 Hz.

Five 1 mm thick transverse slices through the tumour were identified by ^1^H MR scout images. Multi-gradient recalled echo images were then acquired from these slices; a train of eight echoes was acquired with a repetition time of 80 ms, initial echo of 5 ms and an echo spacing of 5 ms, resulting in sets of images with increased *T*_2_^*^ weighting. The total imaging time was ca 14 min. Tumour *R*_2_^*^ (=1/*T*_2_^*^) maps for each slice were generated using all eight gradient-echo image sets by fitting an exponential model on a pixel-by-pixel basis. For each slice, *R*_2_^*^ was determined from an ROI encompassing the whole tumour but excluding the surrounding skin/muscle, and the mean *R*_2_^*^ for each tumour determined from all five slices (Howe *et al*, 1999; Robinson *et al*, 2001). After MGRE MRI, the tumours were excised and assessed blind for necrosis by histology as before.

### Significance testing

The use of MRI for the noninvasive assessment of tumour response to ZD6126 means that each animal serves as its own control. This harnesses significant statistical power through the use of paired tests, while minimising the number of animals required per cohort. Results are presented in the form: mean±1 s.e.m. Significance testing employed the two-sided Mann–Whitney *U*-test for nonparametric data.

## RESULTS

### Dynamic contrast-enhanced MRI study

**Figure 1:**
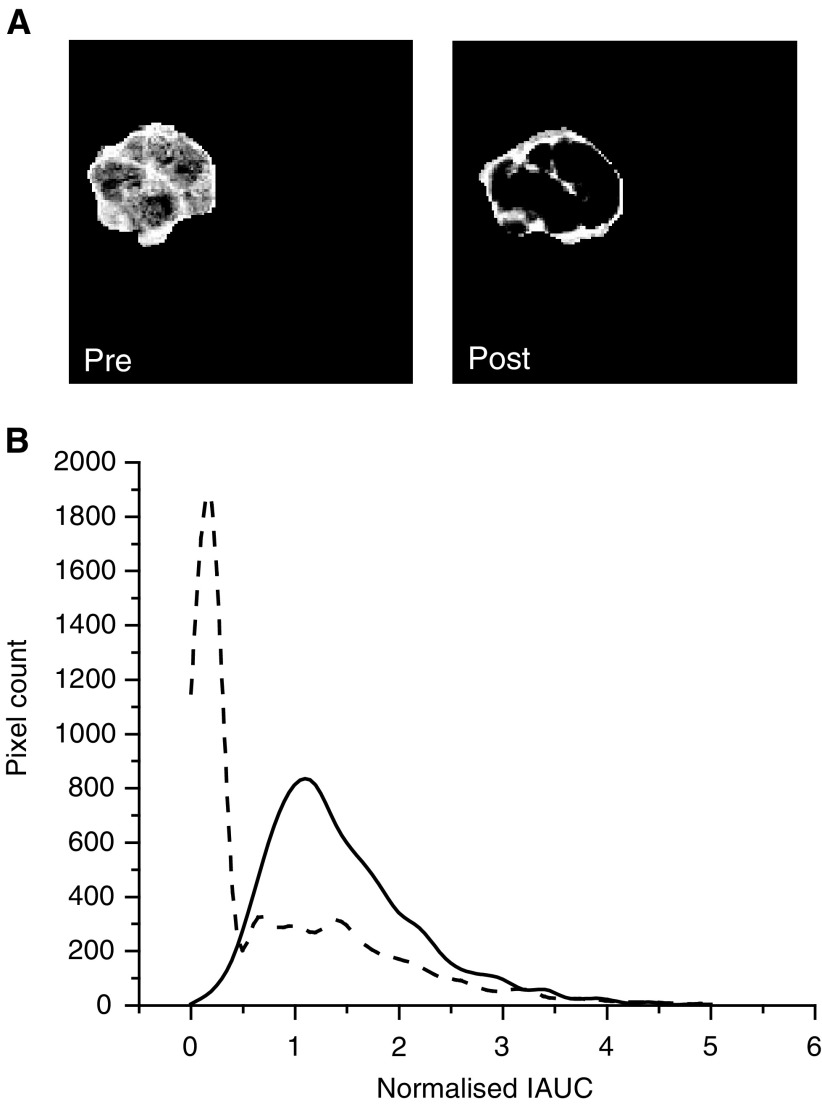
(**A**) Representative *T*_1_-weighted images acquired from one slice of a GH3 prolactinoma prior to and post-treatment with 50 mg kg^−1^ ZD6126. The highly enhancing fraction was clearly limited to the periphery of the tumour post-treatment. (**B**) Normalised IAUC frequency histograms obtained from the same tumour prior to (**---**) and post-treatment with 50 mg kg^−1^ ZD6126 (**–** **–**), acquired as part of the DCE MRI dose–response study. Note the characteristic spike at very low IAUC of the treated tumour group.

Visual inspection of DCE-MRI images showed that the highest dose of 50 mg kg^−1^ ZD6126 essentially eliminated contrast uptake in the centre of GH3 tumour, the highly enhancing tissue being largely confined to the rim 24 h post-treatment. Magnetic resonance images acquired from one representative slice of a GH3 prolactinoma prior to and post-treatment with this highest dose are shown in Figure 1A

. For all the tumours studied, the pretreatment images were heterogeneous, reflecting the heterogeneity in background necrosis in this tumour model (40–50% necrotic fraction). Example normalised IAUC frequency histograms obtained from a rat prior to and post-treatment with the highest dose of 50 mg kg^−1^ are shown in Figure 1B. In the control tumours, a slight, nonsignificant shift towards lower IAUC was apparent, which may reflect tumour growth over 48 h. The between and within subject coefficient of variation of the fraction of highly enhancing pixels in the pretreatment tumours was 26 and 17%, respectively. In all, 14 of the 15 tumours treated with ZD6126 showed a reduction in the highly enhancing fraction. At lower doses of ZD6126, this response was heterogeneous, with some tumour regions being profoundly affected while others appeared spared. At 50 mg kg^−1^ ZD6126, the highly enhancing fraction was limited to the periphery of the tumour post-treatment. This is clearly evident in Figure 1A. Note also the characteristic spike at very low IAUC observed in the frequency histogram of the treated tumour.

Figure 2A

**Figure 2:**
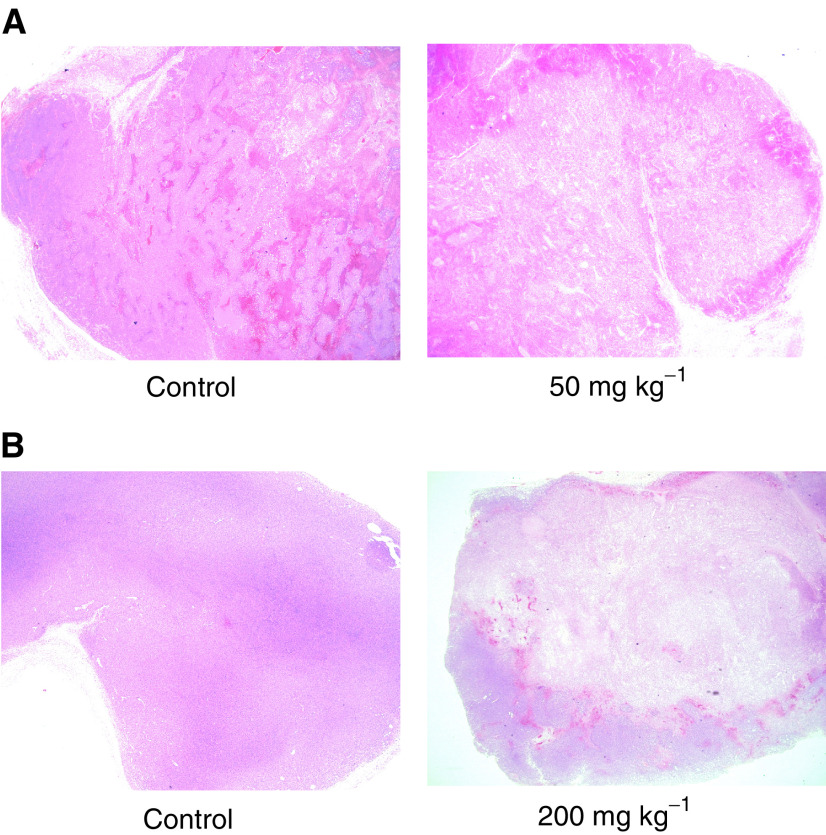
Haematoxylin and eosin stained sections of (**A**) a control and ZD6126-treated GH3 prolactinoma and (**B**) a control and ZD6126-treated RIF-1 fibrosarcoma. Essentially, the darker stain represents more viable tissue. Note that the control GH3 has a larger necrotic fraction compared to the control RIF-1, which is largely viable. ZD6126 caused massive central tumour necrosis, the central necrotic core being surrounded by a viable rim of tumour cells.

shows haematoxylin and eosin stained sections of control and ZD6126-treated GH3 tumours 24 h after drug treatment. Essentially, the darker stain represents more viable tissue. ZD6126 caused massive central tumour necrosis in a dose-dependent manner, the central necrotic core being surrounded by a viable rim of tumour cells.

**Figure 3:**
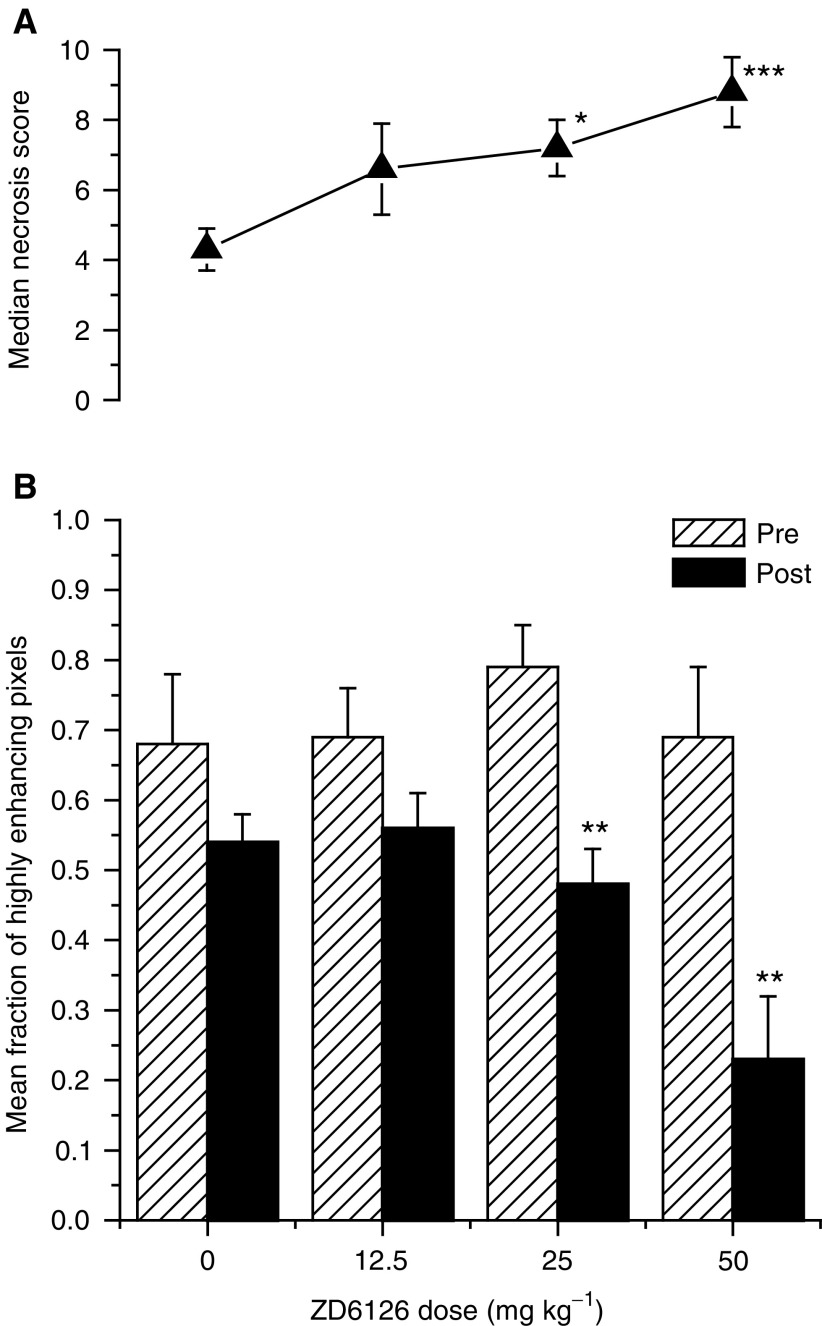
Dose–response of GH3 prolactinomas to ZD6126 assessed by (**A**) necrosis and (**B**) DCE MRI. Treatment with ZD6126 induced a decrease in the mean fraction of highly enhancing pixels in the GH3 prolactinomas in a dose-dependent manner, which was highly significant at 25 and 50 mg kg^−1^ (^**^P<0.02, Mann–Whitney two-tailed *U*-test). Associated with this response was an increase in tumour necrosis in a dose-dependent manner (▴), which was also significant at 25 and 50 mg kg^−1^ (^*^P<0.05, ^***^P<0.01, Mann–Whitney two-tailed *U*-test).

The dose–response of GH3 prolactinomas to ZD6126 is summarised in Figure 3

. ZD6126 induced a decrease in the highly enhancing fraction of GH3 prolactinomas in a dose-dependent manner, and this response was highly significant at 25 and 50 mg kg^−1^. Histological analysis of these same tumours showed that the median necrosis score increased in a dose-dependent manner, significantly at 25 and 50 mg kg^−1^.

### Multi-gradient recalled echo MRI study

**Figure 4:**
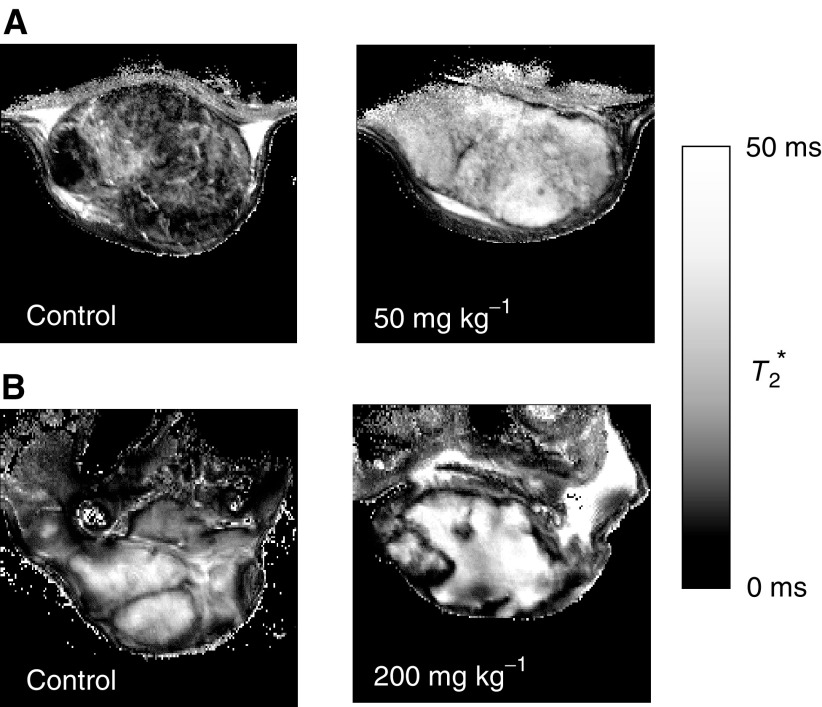
Calculated *T*_2_^*^ maps of (**A**) control and treated GH3 prolactinomas and (**B**) control and treated RIF-1 fibrosarcomas acquired as part of the MGRE MRI efficacy study. *T*_2_^*^ maps, rather than *R*_2_^*^ (=1/*T*_2_^*^) maps, are shown for clarity. Treatment with ZD6126 resulted in an increase in the signal intensity of the *T*_2_^*^ maps of both tumours, consistent with a decrease in tissue [deoxyhaemoglobin].

Calculated tumour *T*_2_^*^ maps from one control GH3 prolactinoma and RIF-1 fibrosarcoma, and one ZD6126-treated GH3 and RIF-1 tumour are shown in Figure 4

. Tumour *R*_2_^*^ (=1/*T*_2_^*^) decreased with treatment. Figure 2B shows haematoxylin and eosin stained sections of a control and ZD6126-treated RIF-1 tumour 24 h after drug treatment. The control RIF-1 tumours were more viable (<10% necrotic fraction) than the control GH3 tumours. In both tumour models, ZD6126 caused massive central tumour necrosis in a dose-dependent manner, the central necrotic core being surrounded by a viable rim of tumour cells (Figure 2). The reduction of *R*_2_^*^ was associated with the induction of extensive central necrosis induced by ZD6126, as measured by histology. The results of this study are summarised in Table 1

**Table 1:** Response of rat GH3 prolactinomas and murine RIF-1 fibrosarcomas to ZD6126, assessed by MGRE MRI and histological assessment of necrosis

Dose (mg kg^−1^)	*R*_2_^*^ (s^−1^)	Necrosis
*GH3 prolactinomas*		
0	78.4±13	5
25	72.6±7	7^#^
50	55.9±9^#^	9^#^
		
*RIF-1 fibrosarcomas*		
0	79.6±23	1
100	64.4±21	9^#^
200	67.2±14	9^#^

Tumour *R*_2_^*^ decreased with treatment (^#^*P*<0.001, Mann–Whitney two-tailed *U*-test), consistent with a decrease in (deoxyhaemoglobin), and which was associated with induction of extensive central necrosis by ZD6126. The necrosis scores represent the median score of three sections for each tumour at each dose level. The median necrosis score significantly increased with treatment in both tumour types (^#^*P*<0.001, Mann–Whitney two-tailed *U*-test).

.

## DISCUSSION

The development and validation of quantitative, clinically applicable MRI end points to assess parameters associated with tumour vasculature is critical for determining the activity of vascular-targeting agents, where access to tumour tissue following therapy is not usually possible. Preclinical models have shown that a single dose of ZD6126 demonstrates significant antitumour activity, causing extensive tumour necrosis, but that this does not necessarily translate into a significant growth delay in human tumour xenografts (Blakey *et al*, 2002; Davis *et al*, 2002). Primarily, this appears to be because of rapid regrowth from viable cells at the rim of the tumour mass. Therefore, noninvasive measures of tumour vascular function are required to examine the activity of ZD6126 in the clinic. Here, we have demonstrated the use of DCE and MGRE MRI to measure the effects of changes in tumour perfusion of transplanted rat GH3 prolactinomas and murine RIF-1 fibrosarcomas, in order to noninvasively assess the dose-dependent tumour vascular-targeting activity of ZD6126. We have also provided validation of these methods by examining the induction of tumour necrosis by histology.

The dose–response of rat GH3 prolactinomas to ZD6126 was measured using DCE MRI and histological assessment of necrosis. Incorporation of a reference muscle tissue permitted an accurate assessment of the tumour IAUC, without direct measurement of the arterial input function. This approach was used to monitor the effects of ZD6126 on the perfusion of GH3 prolactinomas. The proportion of highly enhancing pixels within the tumour was significantly reduced in a dose-dependent manner by ZD6126. These data are consistent with the tumour vasculature being compromised by ZD6126 and subsequent induction of necrosis, which, according to histological analysis, increased in a dose-dependent manner. Lower doses of ZD6126 (12.5–25 mg kg^−1^) reduced the IAUC close to zero within restricted areas of the tumour, typically in the centre of the tumour, while the IAUC remained at pretreatment levels elsewhere. The highest dose (50 mg kg^−1^) reduced the IAUC to zero over the majority of the tumour. This heterogeneous response contrasts with the response seen with other therapeutic approaches to the tumour vasculature, for example, inhibition of VEGF signalling, where the histogram shifts to the left but the shape remains unchanged (Checkley *et al*, 1999, 2001).

Activity of ZD6126 against rat GH3 prolactinomas and murine RIF-1 fibrosarcomas was also evaluated by MGRE MRI. Multi-gradient recalled echo MRI allows the quantification of the transverse relaxation rate *R*_2_^*^ in an efficient and accurate manner. Furthermore, it is completely noninvasive, relying on intrinsic susceptibility contrast mechanisms rather than injection of a contrast agent. An increase in *R*_2_^*^ may reflect an increase in the tissue content of paramagnetic Fe species, primarily deoxyhaemoglobin within erythrocytes (Thulborn *et al*, 1982), consistent with a reduction in tissue perfusion. Conversely, a decrease in *R*_2_^*^ is consistent with increased tissue water associated with oedema or necrosis. We hypothesised that following treatment with ZD6126, haemoglobin within erythrocytes would deoxygenate, resulting in an increase in *R*_2_^*^. This hypothesis was not supported by the data, which showed a decrease in *R*_2_^*^ 24 h after treatment.

Tumour *R*_2_^*^ can be related to tissue vascularity and blood oxygenation by (Howe *et al*, 2001)


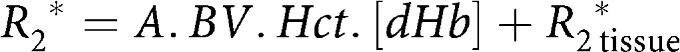


where *A* is a constant dependent on blood vessel size and distribution, *BV* is the fractional blood volume, *Hct* the haematocrit, [*dHb*] the blood deoxyhæmoglobin concentration and *R*_2, tissue_^*^ the intrinsic contribution of the tissue to the tumour *R*_2_^*^. The decrease in tumour *R*_2_^*^ that we measured could be because of one of several factors. ZD6126 induces thrombosis and congestion of erythrocytes in tumour blood vessels 24 h after treatment resulting in extensive central tumour necrosis (Blakey *et al*, 2002; Davis *et al*, 2002). As the erythrocytes coagulate they must become deoxygenated, thereby causing an increase in [*dHb*], and hence an increase in *R*_2_^*^. However, if there is an agglomeration of erythrocytes into small focal areas (Tozer *et al*, 2001), then the average field inhomogeneity could be decreased, causing a reduction in *A* that more than compensates for the [*dHb*] increase, decreasing *R*_2_^*^. There could also be vessel collapse prior to necrosis, which would decrease *R*_2_^*^ via the term *BV*. Lastly, any oedema formed, which may be more fluid than viable tumour tissue, would cause a decrease in *R*_2, tissue_^*^. Although further work is needed to elucidate the exact mechanisms responsible, a change in tumour *R*_2_^*^ measured by MGRE MRI may be a simple and convenient alternative end point for detecting acute changes induced by antivascular therapies.

Histological assessment of ZD6126-treated tumours showed massive necrosis in the centre of the tumour while the peripheral tissue remained viable. This response pattern has been commonly observed in a wide range of different rodent tumour models with both ZD6126 (Blakey *et al*, 2002; Davis *et al*, 2002) and other antivascular therapies (Tozer *et al*, 2001). Despite ZD6126 inducing massive central tumour necrosis alone, the remaining, well-perfused viable rim is the site of tumour regrowth after treatment. Thus, it is likely that ZD6126 will be used in the clinic in combination with conventional therapeutic modalities targeting the tumour periphery, for example, radiation (Siemann and Rojiani, 2001).

In summary, we have shown a strong inverse correlation of decreased IAUC with increased tumour necrosis following treatment with ZD6126. Measurement of IAUC by DCE MRI should provide an unambiguous primary end point of biological activity of antivascular therapies for clinical trial, and also a marker for dose scheduling and timing for adjuvant therapy. As part of the ongoing phase I study of ZD6126, the same DCE MRI approach is being used to measure the antivascular effects in human tumours, and in which an apparent IAUC dose–response effect has been observed (Evelhoch *et al*, 2002). Changes in *R*_2_^*^, measured by MGRE MRI, are complex and require further validation, but may also provide a useful biomarker of response to antivascular therapies. ZD6126 is effective in attacking the tumour vasculature and is a promising agent for the treatment of cancer.
